# Different Faces of Minipuberty in Preterm Twin Girls: A Case Report and Review of the Literature

**DOI:** 10.3390/jcm12020517

**Published:** 2023-01-08

**Authors:** Giorgia Pepe, Mariarosa Calafiore, Maria Rosa Velletri, Domenico Corica, Alessandra Li Pomi, Malgorzata Wasniewska, Tommaso Aversa

**Affiliations:** 1 Department of Human Pathology of Adulthood and Childhood, University of Messina, 98124 Messina, Italy; 2 Neonatology Unit, Bianchi-Melacrinò-Morelli Hospital, 89124 Reggio Calabria, Italy

**Keywords:** minipuberty, hypothalamic-pituitary-gonadal axis, prematurity, gonadotropins, estradiol

## Abstract

Minipuberty (MP) consists of a postnatal activation of the hypothalamic-pituitary-gonadal (HPG) axis, which occurs physiologically during the first months of life. In preterm infants, MP might lead to stronger hormonal stimulation, but specific literature is still scarce. We present the case of a pair of monochorionic diamniotic twin girls, born at 31 weeks of gestation and adequate for gestational age (AGA). At one month old, one of the twins presented with severe edema in the vulva and swelling of the major and minor labia. Laboratory evaluations highlighted increased LH, FSH and estradiol serum concentration. Pelvic ultrasonography and MRI showed a pubertal pattern. Brain imaging was unremarkable. During the one-year follow-up, a decreasing trend of hormonal levels was detected, together with the spontaneous regression of clinical and sonographic pubertal signs. The same hormonal workup was also performed on the other twin, who displayed mildly elevated gonadotropins and estradiol, without evidence of pubertal clinical signs. This case suggests that the amplitude of postnatal HPG activation might be exacerbated in preterm infants, with evidence of puberty changes in clinical, laboratory and sonography data. The spontaneous resolution, together with the exclusion of other causes of precocious puberty, is suggestive for MP of infancy.

## 1. Introduction

Minipuberty (MP) of infancy refers to a transient activation of the hypothalamic-pituitary-gonadal (HPG) axis that occurs physiologically in both sexes during the first months of life [1,2].

In healthy term neonates, FSH and LH rise from 1 week of age and peak between 1 and 3 months, stimulating sex hormone secretion. MP seems to reproduce the same sexual dimorphism previously exhibited in fetal life, with a greater increase in LH in males and FSH in females [3,4]. At approximately 6 months of age, LH and FSH start to decline, but FSH levels could remain high up to the age of 3–4 years in females [5,6]. Likewise, testosterone and estradiol peak at 1–3 months of age in males and females, respectively, and then decrease by 6 months of age [2,6,7,8]. Estradiol levels may sometimes fluctuate until 2 years of age, likely due to ovarian follicle cyclic maturation and atrophy [9,10].

Postnatal HPG activity seems to play an essential role in the maturation of sexual organs and future fertility [1]. Indeed, such activation provides a window of opportunity to examine spontaneous gonadal function before the axis is silenced during childhood. This clinically translates into a golden opportunity for early diagnosis and treatment in infants with suspected hypogonadism [11,12]. Physiologically, MP leads to penile and testicular growth and function through androgen exposure in males, which also predicts the later sex-typed trait (brain masculinization) [13,14]. In females, the biological significance of MP remains more elusive, even though a positive association between estradiol levels and an increase in uterus and breast enlargement has been documented [9,15].

In preterm infants, MP might lead to stronger and more protracted hormonal stimulation compared to full-term babies, but specific literature data are limited and not univocal [9,16,17,18]. Immaturity of the hypothalamic feedback has been suggested as a possible mechanism for this phenomenon, although its biological meaning is still unknown.

We report a case of MP observed in a pair of preterm twin girls with very different clinical expressivity.

## 2. Case Report

A pair of monochorionic diamniotic twin girls, born at 31 weeks of gestation, were admitted to the NICU for prematurity, mild distress respiratory syndrome, anemia and neonatal jaundice. They were born by cesarean section, with Apgar scores of 8–9. They were both adequate for gestational age (AGA) for weight and length (Table 1).

**Figure 1:**
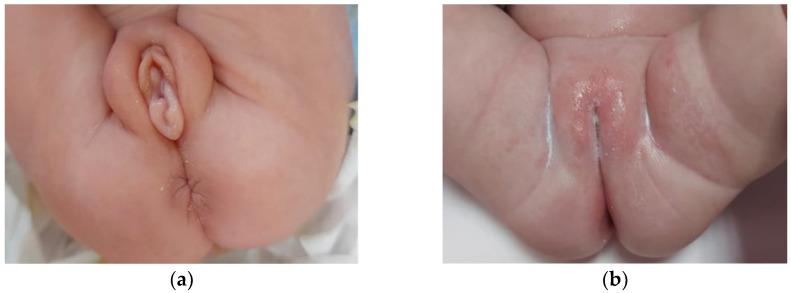
Severe edema of the external genitalia in a preterm twin neonate at 1 month of age (**a**); complete clinical resolution of the pubertal signs observed at 12 months of age (**b**).

At the age of one month, one of the twins (twin A) was referred to our pediatric endocrinology division for the onset of severe edema in the vulva and swelling of the major and minor labia (shown in Figure 1a). No evidence of thelarche, clitoromegaly or clinical signs of pubarche were noticed. At that time, she was on formula feeding, with no medications except for vitamin supplements. Family history was quite uneventful and negative for precocious puberty. No history of any hormonal therapy during pregnancy was reported.

Laboratory evaluations highlighted basal serum levels of gonadotropins in the pubertal range, as well as increased estradiol levels (Table 2). These biochemical results were suggestive for central activation of the HPG axis. Adrenal steroidogenesis and serum electrolytes were within the normal range, thus excluding congenital adrenal hyperplasia. Basal evaluation of the hypothalamic-pituitary-adrenal axis did not show any alterations. Thyroid function, prolactin, alpha-fetoprotein and beta-human chorionic gonadotropin levels were within the normal range. Pelvic ultrasonography (US) and magnetic resonance imaging (MRI) showed increased uterine length (45 mm), endometrial thickness of 4 mm and enlarged ovaries with multiple follicles bilaterally. MRI of the brain and pituitary was unremarkable.

In light of this clinical, biochemical and US picture, we adopted expectant management and monitored the twins throughout the first year of life.

**Figure 2:**
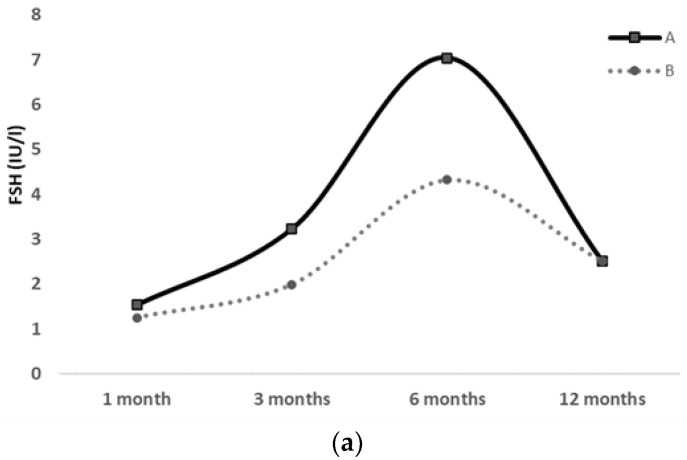

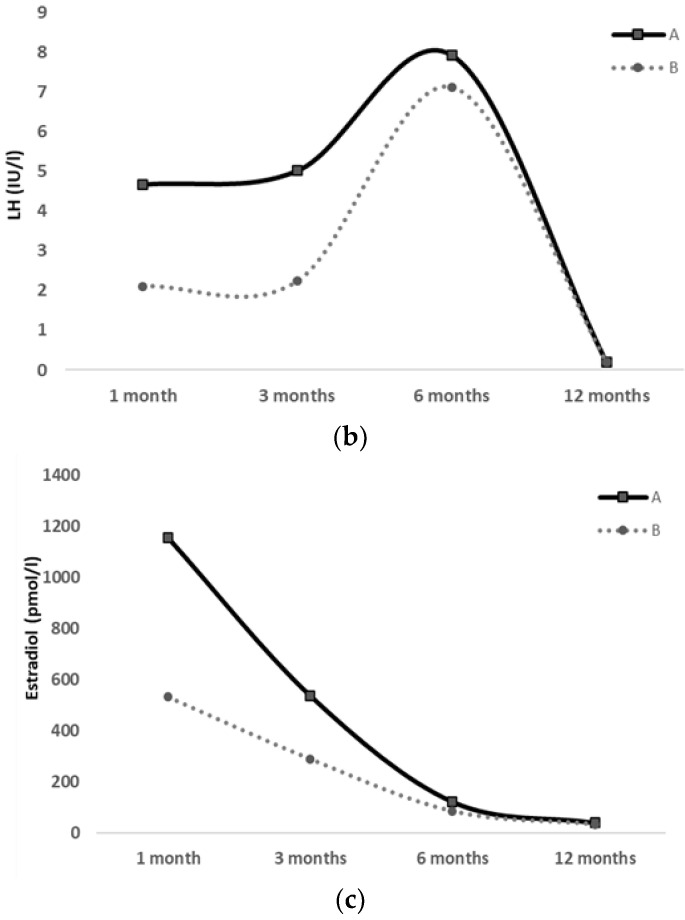
FSH (**a**), LH (**b**) and Estradiol (**c**) serum levels at 1, 3, 6 and 12 months of life in a pair of preterm twin girls; clinical pubertal signs were exhibited only by the first twin (twin A), whereas they were absent in the second twin (twin B) throughout the 12-month follow-up.

Hormonal workup was repeated at 3, 6 and 12 months of age, showing a decreasing trend of gonadotropins and estradiol levels, approximately after 6 months of age (shown in Table 2 and Figure 2).

Clinical and sonographic pubertal signs also spontaneously and gradually regressed over time (shown in Figure 1b).

At the end of the follow-up, we recorded a prepubertal value of LH < 0.1 IU/L and estradiol < 40 pmol/L, together with the complete regression of the vulvar edema. Pelvic US also revealed a more tubular uterus with a fundus to cervical ratio of 1.

The same diagnostic workup was also performed on the second twin (twin B), who showed no evidence of pubertal clinical signs during the 1-year observation period. At 3 months of age, she only displayed mildly elevated levels of FSH, LH and estradiol, which gradually decreased from 6 to 12 months of age (shown in Table 2 and Figure 2). Her US pelvic pattern always presented as prepubertal.

Periodic auxological monitoring highlighted satisfying weight and height gain in both twins during the 12-month follow-up (Table 1).

## 3. Methods

Blood samples were drawn from a peripheral vein in the morning, separated through centrifugation and stored at −20 °C until analysis.

FSH and LH concentrations were reported using international units per liter. Total serum E2 concentration was reported in picomoles per liter. Reference ranges for FSH and LH were based on measurements using time-resolved immunofluorometric assays (Delfia; PerkinElmer, Boston, MA, USA.). The limits of detection (LODs) were 0.05 IU/L and 0.05 IU/L, respectively. The intra- and interassay coefficients of variation were <5% in both assays. E2 was assayed using the commercial RIA method (Estradiol US, Diagnostic System Laboratory, Webster, TX, USA) with threshold of sensitivity of 2.0 pg/mL, and inter- and intraassay coefficients of variation (CVs) were 9.4% and 7.5%, respectively.

## 4. Discussion

According to the data available in the literature, MP also occurs in preterm neonates. In this specific cohort, postnatal HPG activity tends to be even stronger and more prolonged over time than in full-term infants [9,10,16,19]. However, data on MP in preterm vs. term infants are extremely scarce and not conclusive, due to the lack of prospective studies. Consequently, the peculiarity of MP in preterm infants, as well as the clinical implications of such an intensive stimulation, are still not completely understood. Immaturity of the hypothalamic feedback has been suggested as a possible mechanism for this phenomenon, although its biological meaning is unknown [20,21].

In this context, our case report adds new data about peculiarities of MP in preterm newborns, focusing on the different clinical expressivity observed in a pair of monochorionic diamniotic twin girls.

A better understanding of the hormonal changes during the first year of life has been documented by the few most recent longitudinal studies, which highlighted a greater gonadotropin surge in preterm infants if compared to full-term newborns [9,10,16,17,22]. Likewise, estradiol levels seem to be significantly increased in preterm females than in full-term females [10,17,23]. Moreover, a positive association is reported between the postnatal estradiol peak and the enlargement of mammary gland and uterine length. An increase in folliculogenesis after the FSH surge is described as well, and may result in the presence of antral follicles on ultrasonography [9]. This intensive hormonal stimulation may translate clinically into a wide variety of presentations, ranging from thelarche to vaginal bleeding in girls [2,20,24,25] or testicular growth with pubarche in boys [26].

Vulvar edema and labial swelling have also been reported as a consequence of the hormonal stimulation typical of MP, as well as in our patient. This finding was first described by Sedin et al. [27] in the context of ovarian hyperstimulation syndrome in preterm girls. It has also been proposed that edema observed in these subjects is related to vascular endothelial growth factor released from the theca and granulosa cells [28,29]. More recently, other authors reported clinical cases of MP characterized by swelling of the external genitalia observed in preterm girls [30,31].

Furthermore, Kuiri-Hänninen et al. reported sex differences, with preterm females experiencing a more enhanced and prolonged HPG axis activation compared with preterm males. They also observed that postnatal pituitary activity declines at around the same postmenstrual age in premature and full-term newborns, suggesting that the activity of HPG axis is developmentally regulated [2].

More recently, a prospective case–control pilot study was carried out specifically on the evaluation of MP in small for gestational age (SGA) newborns compared with AGA controls, both preterm and full-term. In premature males, both SGA and AGA, higher testosterone and LH levels were detected in the first months of life. Likewise, premature females, both SGA and AGA, exhibited higher estradiol levels than full-term controls [17].

In accordance with these findings, our patients exhibited an increase in LH and FSH serum levels from the first month of life, with a peak at around 6 months, followed by a gradual decrease until normalization by the end of the one-year follow-up. An estradiol peak was recorded earlier, at one month of age, leading to evidence of pubertal clinical signs in one of the twins, who presented with severe vulvar edema and swelling of the major and minor labia. Surprisingly, the second twin never exhibited any clinical presentation during the entire follow-up period, despite her hormonal pattern being suggestive for central activation of the HPG axis as well, even if at a milder level. Our clinical case provided the opportunity to examine MP features in preterm infants, and, therefore, to shed new light on an almost unexplored field. Moreover, the case is peculiar and intriguing in that it showed the discordance of MP presentations observed in a pair of twins, who had been influenced by the same intrauterine and extrauterine conditions. Such a huge variety of clinical and biochemical expressivity of MP was unexpected and challenging to interpret.

After excluding the most important causes of pubertal anticipation, such as central precocious puberty, tumors, congenital adrenal hyperplasia, follicular ovarian cysts and other pathological conditions, we adopted an expectant approach, performing periodic clinical, biochemical and US pelvic monitoring. The spontaneous resolution of symptoms—together with the evidence of the hormonal profile returning to the prepubertal range within 12 months—allowed us to establish the diagnosis of exacerbated MP in preterm infants.

Our clinical case enhances the importance for clinicians of being aware of this physiologic and transient postnatal event, which may be notably exacerbated by prematurity, with evidence of puberty changes in clinical, laboratory and sonography data.

Taking into account the current high rate of premature newborns, clinicians should recognize this phenomenon in the differential diagnosis of precocious puberty, adopt proper management with a conservative approach and, therefore, avoid unnecessary intervention or treatment.

Further prospective studies about MP, especially in premature infants, are needed to allow not only a better understanding of MP, but also to explore the long-term consequences of this hormonal hyperstimulation.

## 5. Conclusions

MP in preterm infants seems to be exacerbated and protracted in time, with evidence in rare cases of puberty changes in clinical, laboratory and sonography signs. The mechanism of this HPG axis hyperactivity in prematurity is not clearly defined, but clinicians should be aware of the self-limiting nature of this phenomenon. Although usually benign, its differential diagnosis includes other causes of precocious puberty. The spontaneous clinical and biochemical resolution, together with the exclusion of other causes of precocious puberty, is suggestive for postnatal HPG axis activation of MP.

## Figures and Tables

**Table 1:** Main auxological parameters assessed in a pair of preterm twin girls (twin A and B) from birth to 12 months of life.

	Birth	1 Month	3 Months	6 Months	12 Months
Weight (g)					
Twin A	1050	1760	3050	4350	8100
Twin B	1210	2200	3620	4880	8800
Length (cm)					
Twin A	36	44	50	58	71
Twin B	38	48	52	59	73

**Table 2:** Biochemical and clinical profile in a pair of preterm twin girls during 12-month follow-up. Comparison between the first and the second twin (twin A and twin B).

	1 Month	3 Months	6 Months	12 Months
LH (mIU/l)				
Twin A	4.66	5.01	7.92	0.2
Twin B	2.10	2.23	7.11	0.2
FSH (mIU/l)				
Twin A	1.53	3.23	7.04	2.5
Twin B	1.25	1.99	4.32	2.5
E2 (pmol/l)				
Twin A	1152.1	535.7	121.69	39.92
Twin B	532.3	289.59	85.49	34.82
Clinical signs				
Twin A	Severe vulvar edema, swelling labia major and minor	Vulvar edema, swelling labia major and minor	Regression of vulvar edema and swelling labia major and minor	absent
Twin B	absent	absent	absent	absent

## Data Availability

The clinical data presented in this study are available on request from the corresponding author.
